# Exploring COVID-19 Phobia among International Chinese College Students in South Korea Before Ending COVID-19 Restrictions

**DOI:** 10.1186/s40359-024-01718-5

**Published:** 2024-04-23

**Authors:** Bo Zhao, Fanlei Kong, Eun Woo Nam

**Affiliations:** 1 Department of Health Administration, Graduate School, Yonsei University, 1 Yonseidae-gil, 26493 Wonju, Gangwon-do Korea; 2 Yonsei Global Health Center, Yonsei University, 1 Yonseidae-gil, 26493 Wonju-si, Korea; 3 Centre for Health Management and Policy Research, School of Public Health, Cheeloo College of Medicine, Shandong University, 250012 Jinan, China; 4 NHC Key Lab of Health Economics and Policy Research, Shandong University, 250012 Jinan, China

**Keywords:** International Chinese College students, Information Trusts, Knowledge-Attitudes-Practices, COVID-19 Phobia, South Korea

## Abstract

**Background:**

College students, considered to be the driving force of society, are highly vulnerable to COVID-19. At a time when facing a new pandemic wave in 2022, China’s policy was in contrast with that of Korea. We investigated the phobia levels of international Chinese college students studying in Korea.

**Objective:**

This study aimed to investigate the relationship between the frequency of use and trust of information sources, and COVID-19 phobia (C19P) among Chinese college students studying in Korea before ending related restrictions.

**Methods:**

This study employed a cross-sectional design, conducting an online survey among Chinese college students studying in Korea from April 8–15, 2022 (before Korea ended the limitations due to COVID-19). Data about 319 respondents were analyzed, including socio-demographics, information variables, knowledge, attitudes, practices (KAP), and C19P. Hierarchical regression analysis with different models was used to examine the relationship between information trust, KAP, and C19P.

**Results:**

Results showed that students performed well in knowledge and preventive practices, had diverse sources of getting information related to COVID-19, and highly depended on the internet and news. Students who perceived a higher severity of infection showed higher levels of COVID-19 phobia. The tendency to wear masks with family/friends, avoid crowded places, and not agree with Korean government mitigation policies reported higher levels of COVID-19 phobia.

**Conclusions:**

More authority and proactive communication strategies, such as consultations or education programs, are needed for international students to alleviate their phobias and psychological stress.

**Supplementary Information:**

The online version contains supplementary material available at 10.1186/s40359-024-01718-5.

## Introduction

Dynamic trends in COVID-19 were unpredictable [1]. With the recurrence of the three-year pandemic, individuals’ psychological experiences were also recurring, leading to problems such as stress, fear, and phobia [2–4]. Previous studies have pointed out that the increased risk factors for mental health and the psychological burden of anxiety and phobia of COVID-19 vary by country [5–7]. Over three-quarters of people surveyed expressed concern for their family members’ health, and more than half of those interviewed reported experiencing moderate-to-severe psychological impacts as a result of their COVID-19 fear [8, 9].

A phobia, unlike regular fear, is a mental health condition that causes an intense fear of specific situations [10]. Thus, a phobia causes anxiety above and beyond the true threat of a scenario, which could have a significant effect on individual’s life [11]. Studies indicated that the phobia of COVID-19 is an exaggerated fear of contracting the virus causing COVID-19, accompanied by excessive concern about physiological symptoms, increased stress and safety-seeking behaviors and avoidance of public places and situations, resulting in a noticeable impairment in daily functioning [12, 13]. These worries, combined with social isolation and strict quarantine regulations around the world, have resulted in unparalleled stress on individuals, both mentally and emotionally. This uncontrollable and persistent fear has given rise to a new and distinct type of anxiety known as COVID-19 phobia (C19P), which has exacerbated the effects on mental health [8, 9].

The health and well-being of vulnerable groups during the COVID-19 pandemic need to be prioritized [5–7]. College students have long been regarded as one of the most vulnerable groups to psychological health disorders [14–16]. As well-educated young adults and a group that has active social lives and great social mobility, college students were at a high risk of being exposed to and infected with COVID-19 [17, 18]. For international college students abroad [19], the COVID-19 pandemic led to extra pressure and challenges for those who were away from central social support such as family and friends [20]. The COVID-19 psychological and mental well-being studies of international students from different countries have been conducted in several countries [21–23], revealing there was increased phobia and urgency for further care concerning international students’ psychological status.

Regards to the Korea Education Development Agency, 67,439 Chinese students were studying in South Korea, accounting for 40.4% of the international student population in 2022 [24, 25]. Previous studies mentioned that some international students who may lack proficiency in the domestic language and cultural customs prefer interacting with people from their home culture and may avoid getting involved in extracurricular activities. Due to the social limitations of COVID-19, online classes were chosen and they did not need to go to school to meet other people. Faculties also highlighted the difficulty in increasing involvement among local Korean students and international students during the COVID-19 pandemic. Thus, there were rare meaningful opportunities for socialization for Chinese international students, leading to a struggle to adapt to Korean life [25].

According to previous studies, the factors affecting college students’ phobia of COVID-19 could be divided into two aspects individual and social relations. Individual factors included age, gender, educational level, living area, COVID-19 isolation experience, knowledge, avoidance behavior, and trusting government measures and information [26, 27]. COVID-19 knowledge could directly affect attitudes (e.g., perceived risk of infection) and practices (e.g., social distancing and personal hygiene practices), which were associated with C19P and have been studied by numerous researchers [28–30]. Although variations in the level of knowledge-attitude-practice (KAP) and phobia of COVID-19 exist among different countries and groups [29, 30], increasing knowledge, building positive attitudes, and improving behavioral patterns have been suggested to assuage people’s phobia and stress of successful COVID-19 control [31, 32].

Internet and social media have emerged as primary methods for residents to obtain dynamic information and maintain social contact and relations [33, 34]. The implementation of home isolations and restrictions of in-person social interactions severely restricted public outings and social interactions on a broad scale [35, 36]. The an extraordinary amount of misinformation regarding the pandemic [37, 38]. This has created a fertile atmosphere for fake news or misleading information to propagate, preying on people’s helplessness, unawareness, and restlessness [39, 40]. For example, lack of information on COVID-19 and acquiring wrong information from their peers are associated with the onset of panic and phobia among young teenagers. College students with higher levels of frequency use of information sources or trust could have a higher risk of phobia of COVID-19 [41]. What is more concerning is that many Internet users may be incorrectly receiving or interpreting information, thereby increasing the spread of inaccurate information [42, 43]. Consequently, exposure to trustworthy internet channels may favorably expose one to dread [44] and generate a panic reaction surrounding COVID-19.

In 2020–2021, Korea successfully contained COVID-19 and was hailed as an example for other countries to learn from [45, 46]. However, in March 2022, the number of daily new cases in Korea was more than 407,000, with one in five South Koreans being infected with COVID-19 [47]. With the increase of daily confirmed cases (3rd wave), at the beginning of April, South Korea stated that it would further relax its social distancing rules and eliminate most pandemic-related regulations [48, 49]. Starting on April 18, 2022, the Korean authorities agreed to suspend daily sanitary regulations, except for the mask mandate [50]. During the same period, the severity of COVID-19 in Shanghai, China, showed no signs of easing, which led other Chinese cities to tighten curbs, even in places with no recent infections [51]. Such a considerable difference in the two countries’ views and practices on COVID-19 may bring more restlessness and phobia to Chinese international students studying in South Korea. Although many studies explored the mental status among international student groups, there were rare studies that have examined the potential phobia of COVID-19 among Chinese international students in Korea during a totally different government policy.

Therefore, the following hypothesis was developed: C19P of college students may be affected by individual factors, information factors, and KAP aspects. This study aimed to investigate the level of COVID-19 knowledge, trust in information sources, KAP, and phobia of COVID-19 among Chinese international students studying in Korea during the COVID-19 3rd wave. Additionally, under the different COVID-19 regulations in the home country and host country, it aims to investigate the influencing factors of COVID-19 phobia among these students and tentatively explore the impact of the Korean mitigation policy opinion on phobia in 3rd COVID-19 period.

## Methods

### Study design and sample size

**Fig. 1:**
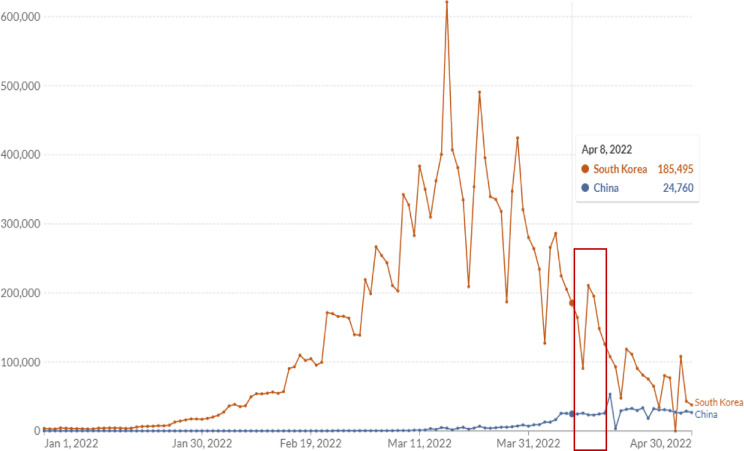
Daily new confirmed COVID-19 cases in South Korea and China in 2022 [52]. *Note* Red box: survey period

In this cross-sectional study, we collected data from respondents using an anonymous questionnaire. Snowball sampling was used to recruit participants. An online questionnaire made from an online platform (Wenjuan.com) was distributed through WeChat among Chinese international college students. The survey started on April 8, 2022, and ended on April 15, 2022 (Fig. 1), recruiting a total of 319 participants.

The sample size needed for the study was calculated using the G*Power 3.1 program with choosing F test for multiple regression analysis based on the study design, a moderate effect size (f^2^ = 0.15), an alpha-level of 0.01, a power of 0.95 and 9 predictors [53]. The minimum total sample size was estimated to be 158. Considering the population of this group, the sample size were also calculated by the determinants and prevalence of psychological issues brought on by COVID-19 [41, 54]. Based on the following single population percentage formula assumptions: 5% type I error, 95% CI and 80% response distribution, then the final sample size became 247.

$$ n=\frac{(Z{\alpha }/2)2\left(P\right)(1-P)}{d2}$$

where n = required sample size, Zα/2 = critical value for normal distribution at 95% confidence level (1.96), P = proportion of psychological problems and d = 0.05 (5% margin of error).

Chinese college students studying in Korea were sent a link to participate in the questionnaire. They were invited to complete the survey online and were encouraged to send the link to their acquaintances. All participants were informed the statement of the purpose of the research and assurance of the confidentiality and privacy. Students could only complete the questionnaire after reading this statement and click-ing “AGREE” to confirm their consent. Additionally, the main questions in the survey were stipulated as mandatory questions. Any question left blank made the final submission of the online questionnaire impossible. All these measures resulted in a 100% response rate for our study.

### Measurements

Scales for measuring information trust, the KAP, and COVID-19 phobia have been developed in previous studies. The Yonsei Global Health Center (YGHC) has made some changes according to the specific situation in Korea. The questionnaire in this study consisted of questions covering several areas: (1) demographic characteristics, (2) COVID-19 experiences and information, (3) KAP of COVID-19, and (4) COVID-19 phobia.

- Demographic characteristics elicited from the respondents included gender, age, educational level, living areas, living status, and religion.
- COVID-19 experiences and information included vaccination status, whether the student had COVID-19, mitigation opinion, information-related sources, and trust. Questions on seven information sources, including Internet/TV news, family/fiends, hospital/medical personnel, social media/SNS, public institutions, phone, and radio, were asked to respondents about using frequency who answered on a 5-point Likert-type scale (“1 = never, 5 = always”). Questions on the trust scores (1–10) of the media, public, and family/friends were subsequently asked (Table 1).
- The KAP scale was adopted from similar studies [55] and validated by Lee et al. [56]. Knowledge of COVID-19 was assessed using a six-item questionnaire developed by Zhong et al. [57]. All respondents could respond with “Yes,” “No,” or “Don’t know.” Knowledge scores were calculated by assigning one point to each correct question, and an aggregate score was calculated (range 0–6), with higher scores indicating more knowledge about COVID-19. Attitudes related to COVID-19 were examined, including the perceived risk of COVID-19 infection and perceived severity of infection (two items: if COVID-19 infects you, if COVID-19 infects family/friends) [58]. Responses were rated on a 5-point Likert-type scale, with “1 = very low, 3 = neither low nor high, and 5 = very high.” Precautionary behavior practices were measured using five items in two categories: (1) preventive measures (i.e., wearing facial masks in public/with family or friends and practicing internal ventilation regularly) and (2) social distancing (i.e., avoiding crowded places and maintaining social distancing). Respondents were asked to give scores on their practices ranging from to 1–10 (1 = not at all and 10 = extremely) (Table 1).
- The COVID-19 Phobia Scale (C19P-SE) was developed, and its reliability and validity were confirmed by Arpaci et al. [59, 60]. The C19P-SE purports to assess phobic reactions using 20 items followed by 4-factor categories (psychological, psychosomatic, economic, and social scale). The items in the Appendix are graded on a five-point Likert scale ranging from “strongly disagree (1)” to “strongly agree (5).” The total scale scores range from 20 to 100, with higher scores indicating more phobia. The total scale in this study had a Cronbach’s value of 0.940 and Kaiser-Meyer-Olkin (KMO) of 0.936. The reliability and validity of the subscales were tested (Table 2).

**Table 1:** Descriptive statistics of COVID-19 phobia scale (C19P-SE) (*N* = 319)

Scale	Items	Range	Mean ± SD	α	KMO
**C19P-SE**	20	20–100	56.47 ± 17.64	0.940	0.936
Psychological	6	1–30	19.14 ± 6.01	0.855	0.876
Psycho-somatic	5	1–25	9.60 ± 4.91	0.874	0.840
Economic	5	1–25	17.08 ± 5.07	0.816	0.788
Social	4	1–20	10.62 ± 1.26	0.836	0.772

**Table 2:** Characteristics of study participants (*N* = 319) and their mean score of C19P-SE

Variables	Total	C19P-SE	*P*
	n (%)	Mean ± SD	
**Total**		56.47 ± 17.64	
**Age** ^a^	26.53 ± 4.60		
**Gender**			0.048
Male	111 (34.8)	53.66 ± 19.37	
Female	208 (65.2)	57.97 ± 16.49	
**Education level**			0.372
Undergraduate and others (e.g.: language learning program)	66 (20.7)	58.45 ± 21.05	
Graduate and above	253 (79.3)	55.95 ± 16.64	
**Major type**			0.015
Medical/health-related	34 (10.7)	49.50 ± 15.78	
Non-medical	285 (89.3)	57.30 ± 17.69	
**Religion**			0.083
Have religion	40 (12.5)	51.95 ± 20.06	
No religion	279 (87.5)	57.11 ± 17.20	
**Living alone**			0.673
Yes	162 (50.8)	56.06 ± 17.42	
No	157 (49.2)	56.89 ± 17.90	
**Living area**			0.442
City	295 (92.5)	56.26 ± 17.58	
Rural	24 (7.5)	59.04 ± 18.51	
**Vaccination**			0.915
Not vaccinated	8 (2.5)	57.13 ± 21.60	
At least one dose	311 (97.5)	56.45 ± 17.57	
**Got COVID-19 or not**			0.020
Yes	33 (10.3)	49.73 ± 16.32	
No	286 (89.7)	57.24 ± 17.64	
**Government’s COVID-19 mitigation policy opinion** ^**b**^			< 0.001
Do not agree	196 (61.4)	62.63 ± 15.41	
Do not care	70 (21.9)	46.90 ± 14.62	
Agree	53 (16.6)	46.32 ± 18.89	
**COVID-19 Phobia level**			
Low (20–40)	57 (17.9)	31.53 ± 6.12	
Medium (41–70)	192 (60.2%)	54.73 ± 7.98	
High (71–100)	70 (21.9)	81.53 ± 8.21	

*Note*^a^: mean age; ^b^: ANOVA test

### Statistical analysis

Descriptive analysis, Student’s t-test or one-way analysis of variance (ANOVA) was used to assess statistical differences in the distribution of COVID-19–related degrees of phobia across different sociodemographic factors. The association between trust in information sources, KAP, and phobia of COVID-19 was investigated using multivariate linear regression hierarchical model.

The correlation magnitudes are reported as standardized regression coefficients (β). SPSS, version 23.0 (IBM Corp) was used to perform statistical analyses. VIF ranged from 1.02 to 2.50, indicating that there was no multi-collinearity among selected independent variables. Different model regression results showing in the Forest plot were graphed by GraphPad prism 8 (GraphPad Software, Inc., San Diego, CA). All statistical significance levels were set at α = 0.05 (*P* < 0.05), and all statistical tests were two-tailed.

The inclusion criteria were as follows: (a) college students aged over 18 years old, (b) Chinese students studying at Korean universities, and (c) consent to participate in the survey. In total, 319 valid responses were included in the data analysis.

## Results

### Participant characteristics

The mean age of the participants was 26.53 years (SD = 4.60). Of the 319 participants, 65.2% were women, and 34.8% were men. Of these, 253 (79.3%) were in graduate or above educational status, while only 34 (10.7%) majored in medical/health-related. Most of them (87.5%) did not have a religion, and half of them were living alone. More than 90% of these students lived in city area and got vaccinated at least one dose. 33 out of 319 participants (10.3%) were COVID-19 positive, and around 61.4% did not agree with Korea’s mitigation policy. The mean COVID-19 phobia score was 56.47 (SD 17.64). More than half (60.2%) had a medium phobia level. Significant differences in phobia scores were reported in groups of major, get COVID-19, and government’s COVID-19 mitigation policy (as shown in Table 3).

**Table 3:** Responses of Information sources, trust, and KAP (*N* = 319)

Variables	Range	Mean	SD
**Frequency of information sources use**			
Information sources frequency score	7–35	21.10	4.30
**Information trust**			
media trust: how much do you trust the COVID-19 information provided by the media (Internet/TV/SNS)?	1–10	7.15	1.34
public trust: how much do you trust the COVID-19 information provided by public health centers/public institutions?	1–10	7.50	1.90
family/friends trust: how much do you trust the COVID-19 information provided by your family/friends?	1–10	6.74	2.02
**Knowledge**			
Knowledge score	0–6	5.07	1.14
**Attitudes**			
*Perceived risk*			
Perceived susceptibility	1–5	3.28	1.22
Perceived severity (you)	1–5	2.56	1.06
Perceived severity (family/friends)	1–5	2.87	1.04
**Practices**			
P1: Wearing facial masks (public places)	1–10	9.59	0.93
P2: Wearing facial masks (with your family/friends)	1–10	3.19	3.20
P3: Practicing internal ventilation regularly	1–10	9.42	1.38
P4: Avoiding crowded places	1–10	8.43	2.02
P5: Keeping social distancing	1–10	9.77	0.92

**Fig. 2:**
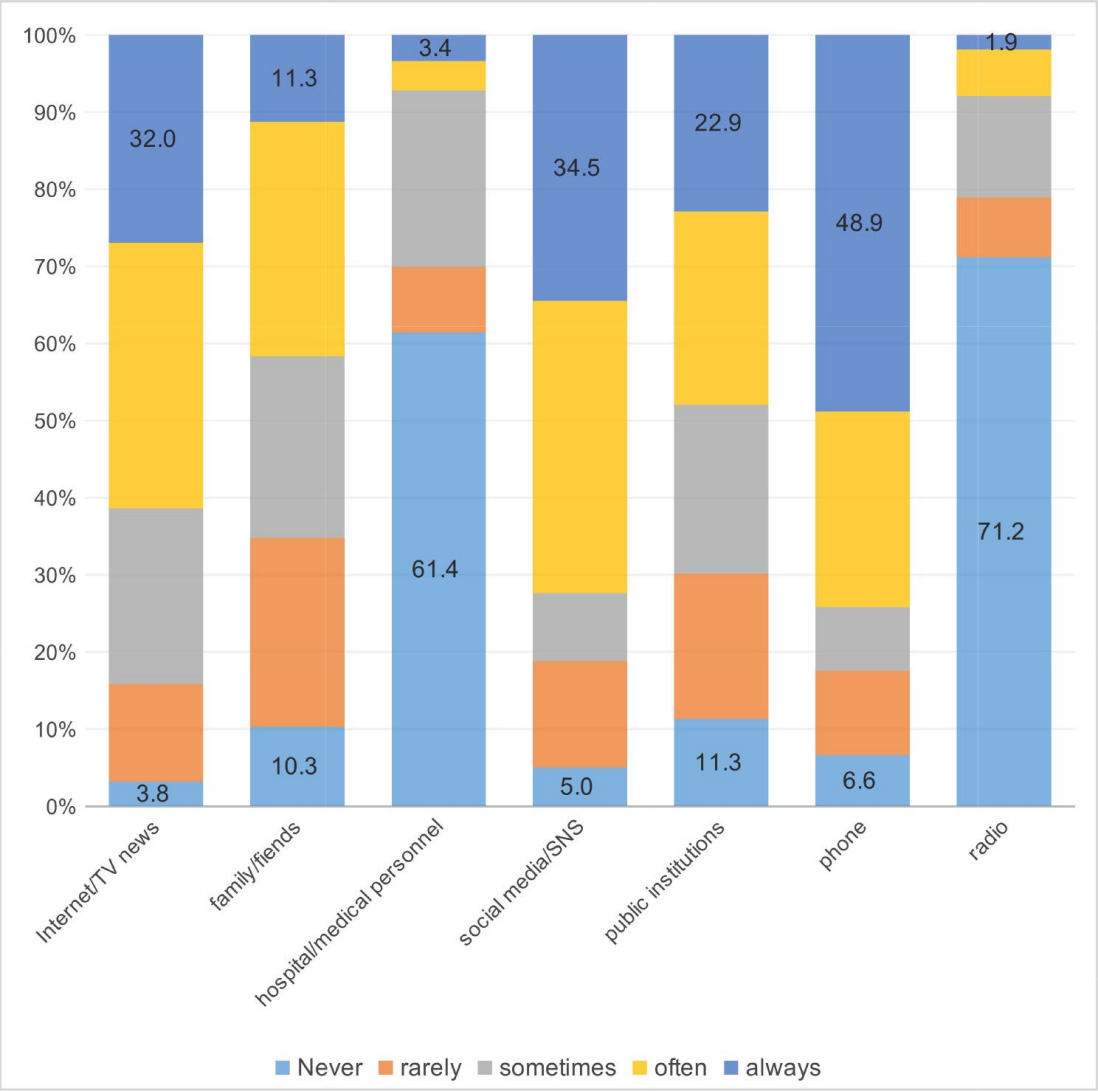
The percentage of responses to frequency of information sources items usage (*N* = 319, %)

Figure 2 shows the usage frequency of seven information sources among Chinese college students studying in Korea. Nearly half (48.9%) of them always used phones to obtain information, followed by social media (34.5%), internet/TV news (32.0%), and public institutions (22.9%). Additionally, the sources that most of them had never used included radio (71.2%) and hospital/medical personnel (61.4%). The total information source frequency scores of these respondents were 21.10 ± 4.30 (Table 1).

**Fig. 3:**
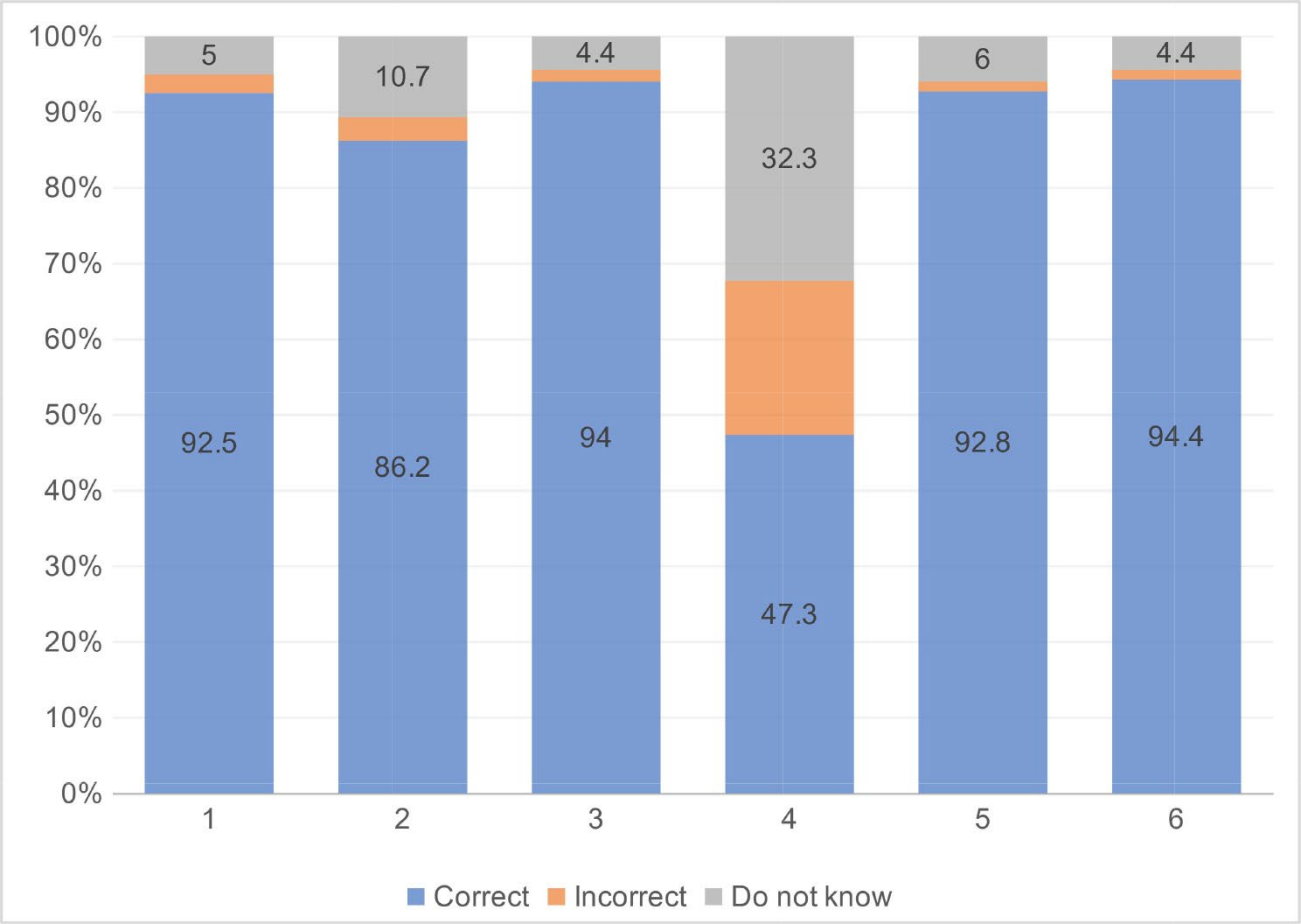
The percentage of responses to knowledge items (*N* = 319, %) *Note 1*: *The main clinical symptoms of COVID-19 are fever, fatigue, dry cough, and myalgia. 2: There is currently no effective cure for COVID-2019, but early symptomatic and supportive treatment can help most patients recover from infection. 3: Not all persons with COVID-2019 will develop severe cases. Only older individuals who have chronic illnesses are more likely to be in severe cases. 4: Eating or contacting wild animals would result in infection by the COVID-19 virus. 5: The COVID-19 virus spreads via respiratory droplets of infected individuals. 6: Ordinary residents can wear general medical masks to prevent infection by the COVID-19 virus.*

Most respondents answered approximately five of the six knowledge items correctly (M = 5.07, SD = 1.16). These students appeared to be knowledgeable about transmission through the respiratory droplets of infected people (92.5% answered correctly, 2.5% incorrectly, and 5.0% reported that they did not know). A high prevalence of misunderstanding was discovered in a knowledge item, with participants believing that infection could occur through eating or having contact with wild animals (Fig. 3). Only 20.4% correctly answered that the statement was false, 47.3% believed it was true, and 32.3% said that they were not sure. Around 94.4% of the respondents replied that wearing a general medical mask helped prevent the spread of infection. The mean total knowledge score was 5.07 ± 1.14 (Table 1).

Respondents trusted the information provided by public health centers and institutions (M = 7.50, SD = 1.90), followed by the Internet/TV (M = 7.15, SD = 1.34), and family/friends (M = 6.74, SD = 2.02). They perceived the risk of becoming infected with COVID-19 (perceived susceptibility) as higher than “neither high nor low” (score = 3) (M = 3.28, SD = 1.22). The average perceived severity scores in “yourself” and “family/friends” were average level (M = 2.56, SD = 1.06; M = 2.87, SD = 1.04). Four out of five practice items were reported well. The most frequently performed practice was maintaining social distancing (M = 9.77, SD = 0.92), followed by wearing facial masks (M = 9.59, SD = 0.93) in public places, practicing regular internal ventilation (M = 9.42, SD = 1.38), and avoiding crowded places (M = 8.43, SD = 2.02) (Table 1).

### Associations between demographic variables, Information trust, KAP and phobia of COVID-19

**Fig. 4:**
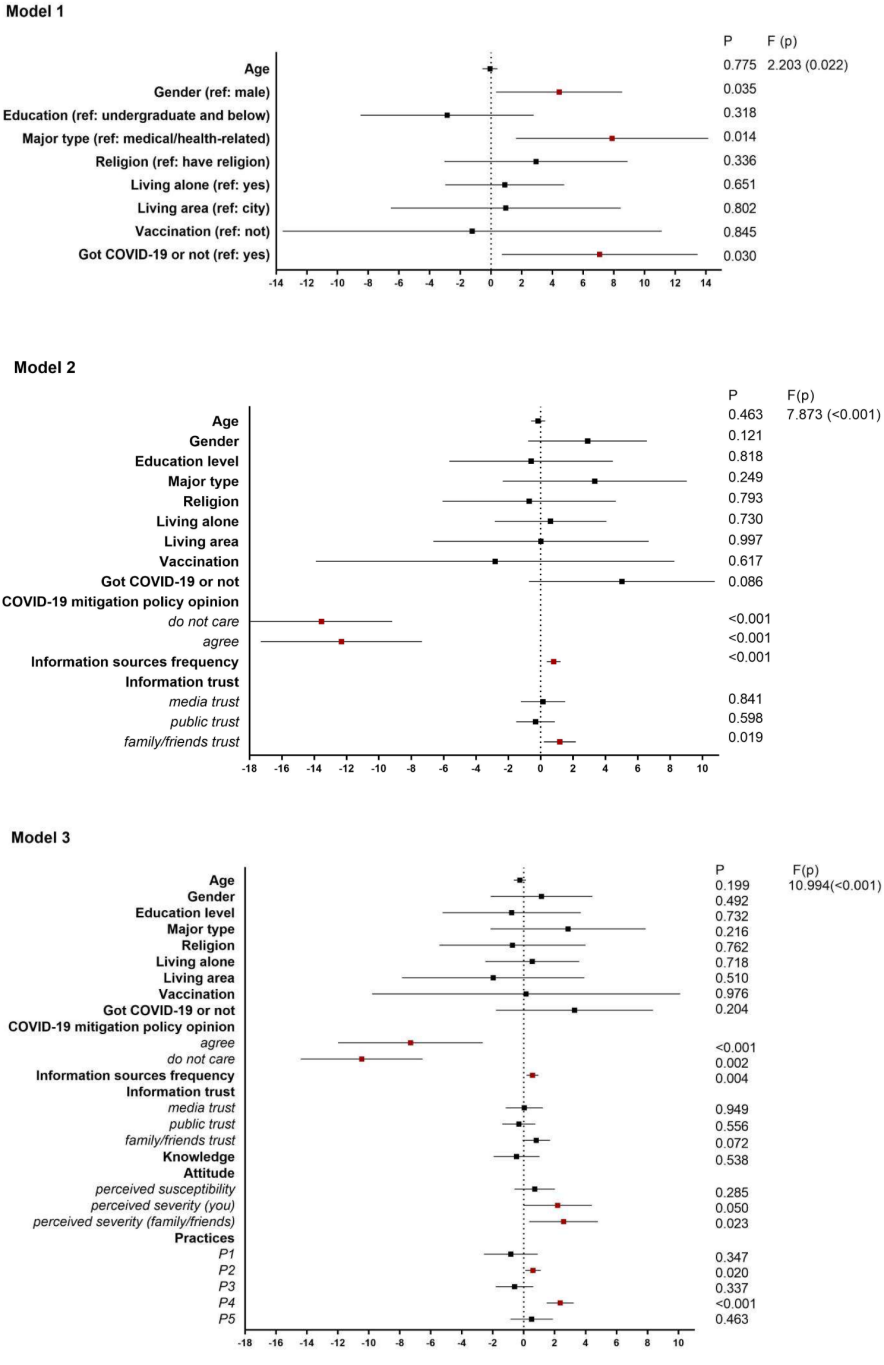
Regression analysis of the phobia of COVID-19 among international Chinese college students in Korea (*N* = 319). *Note* Significant symbols are marked in red (*P* < 0.05)

Figure 4 shows that gender, major type, and COVID-19 were significantly associated with COVID-19 phobia (Model 1). Women with a higher level of education who did not have COVID-19 before were more likely to have severe phobia. After adding information-related variables in Model 2, it was reported that those who did not agree with the mitigation policy, who more frequently received information, and who trusted their family would have a greater risk of phobia. Model 3 shows more associations with the KAP variables. Perceived higher severity of infection, better performance in wearing masks with family/friends, and avoiding crowded places were also significantly associated with a higher level of phobia. The Durbin-Watson value was 2.092 and the F-test in the three models was less than 0.05. The R^2^ value accounted for 47.3% of the variation in Model 3.

## Discussion

### Principal results

Our study demonstrated the level of trust and frequency in information sources, KAP, and COVID-19 phobia among Chinese international students in South Korea. In addition, we examined whether trust and frequency in information sources and KAP were associated with phobia of COVID-19. In the context of Korea’s ending restrictions on COVID-19, this is one of the few studies that considers the relationship between these factors for this special population group.

This study reported that the respondents had diverse sources of getting information related to COVID-19. After two years of knowledge of COVID-19, they had adequate knowledge about COVID-19, including the transmission of the virus through respiratory droplets of infected people and clinical symptoms of the disease. Information trust via the media and the public was higher than that in family or friends. Regarding attitudes, the perceived risk of infection susceptibility was relatively higher than the disease’s perceived severity, which was the opposite in a 2021 Korean group study [56]. More than half of them disagreed with the mitigation policy, which could be explained by the fact that China’s behavior affected their opinion, which has been escalating restrictions [61, 62]. Most respondents complied with the recommended practices, such as wearing facial masks in public, practicing regular internal ventilation, avoiding crowded places, and maintaining social distancing to prevent COVID-19 infections. Although the KCDC said that if a family member/roommate becomes infected, others can change to self-manual surveillance and go out normally [63], and they do not score high on masks when they are with family/friends. This may be because most of them live alone and do well to avoid meeting others [64, 65].

Given that individuals need to learn about the disease and then respond properly, health information acquisition and sharing have become increasingly vital during the COVID-19 pandemic [66, 67]. Information from highly trusted sources is more likely to elicit changes in action and mental status. Health-related information can be provided through a series of interpersonal, organizational, and mediated communication channels (e.g., health personnel, public agencies, and social media) [68, 69]. For example, the daily regional COVID-19 situation can be noticed by people through phones in Korea. A higher frequency of use and trust in COVID-19 information sources provided by family/friends were associated with increased phobia of COVID-19 among these students, according to the findings. This might indicate that more COVID-19 information is exposed and accepted by those who are highly supported by their family and friends [70], and they felt greater fear as a result of misinformation or fake news such as rumors and disinformation, which might lead to several panic actions [71, 72]. This may be an unintended consequence of providing COVID-19 messages daily through many platforms [70]. A previous study also reported that misinformation regarding COVID-19 shared on social media may have caused unwarranted fear, anxiety, and stigma against affected individuals [73]. These international college individuals had disagreed with Korea’s mitigation policy, and there were too much information about China and Korea for them to filter out helpful information or assess their personal dangers [74]. Further assurances about their safety or interpretations of official messaging on social media and public agencies may make them feel less fearful.

The results of the regression analysis on the association between KAP and COVID-19-related phobia support the findings of previous research on the relationship between KAP and fear of COVID-19 [29, 30, 75]. Levels of knowledge, risk perception, and preventive health behaviors during the COVID-19 outbreak have been identified as essential adherence elements for implementing prevention and control measures [76, 77]. In this study, instead of knowledge, Chinese international students’ emotions related to panic and fear could be more affected by attitudes and preventive practices. Being at high risk of coming into contact with confirmed patients without any limitations while the home country’s situation was getting worse and escalated, the policy gap and pandemic situation of the two countries exposed the vulnerability of international students in Korea [78, 79]. More perceived severity of family/friends getting COVID-19, wearing masks with family/friends, and avoiding crowded places were significantly associated with higher phobia. Psychological elements, such as fear and phobia, must be considered in infectious disease treatment and surveillance [80]. The best way to relieve the phobia of COVID-19 among Chinese international college students may be to focus on communication from authority standpoints to remove deep-seated beliefs and misperceptions.

The study’s findings offer both theoretical and practical implications. Theoretically, it contributes to understanding how information sources and social support networks impact emotions during pandemics, particularly phobia among international students. Practically, it highlights the need for accurate, authoritative communication to manage COVID-19 misinformation and its psychological effects. Tailoring communication strategies to address misconceptions and providing clear, reliable information can mitigate phobia and support better adherence to preventive measures among vulnerable populations like international students.

## Limitations

This study has certain limitations. First, the non-probability sampling approach used in the data collection may restrict readers from generalizing the findings to larger settings. However, it is routinely used in social and medical sciences when the target population is difficult to identify [81], particularly during such a unique pandemic. Therefore, conducting a generalizable study with the help of platforms and institutions among possible participants using probability methods such as random sampling and increasing the sample size would be ideal. Second, the assessment items for information, KAP, were examined and subjectively answered in this study. Attitudes related to COVID-19 risk perceptions could include not cognitive but also affective dimensions. Additional characteristics linked to COVID-19 attitudes and preventive behaviors, such as perceived obstacles or other communication factors that may have impacted their phobia levels. Third, comparable study with Korean or other international students to better understand the role of nationality in phobia would be future planning in the similar research. Nevertheless, the strength of this study is that results from Chinese international students in Korea about information trust, KAP, and COVID-19 phobia will be directly applicable to the new normal after ending all COVID-19 limitations.

## Conclusions

The findings from this study suggest that Chinese international students who highly trusted family-provided information, more frequently used sources to get information, and disagreed with Korea’s mitigation policy had higher levels of phobia of COVID-19. In the context of ending all limitations and returning to normal, there was also evidence of a strong association between the perceived severity of family/friends getting infected, preventive practices, and the increasing risk of COVID-19 phobia among these students. These results have implications for mental interventions for COVID-19, suggesting the need for more proactive communication strategies and education programs for international students to alleviate their phobia and psychological stress.

### Electronic supplementary material

Below is the link to the electronic supplementary material.

Supplementary Material 1

## Data Availability

The datasets generated and/or analyzed during the current study are not publicly available but are available from the corresponding author on reasonable request.

## References

[1] Kim K, Lee M (2021). The impact of the COVID-19 pandemic on the unpredictable dynamics of the cryptocurrency market. Entropy.

[2] Chevance A, Gourion D, Hoertel N, Llorca PM, Thomas P, Bocher R, Moro MR, Laprévote V, Benyamina A, Fossati P, Masson M (2020). Ensuring mental health care during the SARS-CoV-2 epidemic in France: a narrative review. L’encephale.

[3] Jackson L, De Pascalis L, Harrold JA, Fallon V, Silverio SA (2021). Postpartum women’s psychological experiences during the COVID-19 pandemic: a modified recurrent cross-sectional thematic analysis. BMC Pregnancy Childbirth.

[4] Han C, Li M, Haihambo N, Babuna P, Liu Q, Zhao X, Jaeger C, Li Y, Yang S (2021). Mechanisms of recurrent outbreak of COVID-19: a model-based study. Nonlinear Dyn.

[5] Rahman MA, Islam SM, Tungpunkom P, Sultana F, Alif SM, Banik B, Salehin M, Joseph B, Lam L, Watts MC, Khan SJ (2021). COVID-19: factors associated with psychological distress, fear, and coping strategies among community members across 17 countries. Globalization Health.

[6] Banerjee D, Vaishnav M, Rao TS, Raju MS, Dalal PK, Javed A, Saha G, Mishra KK, Kumar V, Jagiwala MP (2020). Impact of the COVID-19 pandemic on psychosocial health and well-being in South-Asian (World Psychiatric Association zone 16) countries: a systematic and advocacy review from the Indian Psychiatric Society. Indian J Psychiatry.

[7] De Kock JH, Latham HA, Leslie SJ, Grindle M, Munoz SA, Ellis L, Polson R, O’Malley CM (2021). A rapid review of the impact of COVID-19 on the mental health of healthcare workers: implications for supporting psychological well-being. BMC Public Health.

[8] Marzo RR, Ismail Z, Htay MN, Bahari R, Ismail R, Villanueva EQ, Singh A, Lotfizadeh M, Respati T, Irasanti SN, Sartika D (2021). Psychological distress during pandemic Covid-19 among adult general population: result across 13 countries. Clin Epidemiol Global Health.

[9] Lahav Y. Psychological distress related to COVID-19–The contribution of continuous traumatic stress. J Affect Disord. 2020;277. 10.1016/j.jad.2020.07.141.:129– 37. 10.1016/j.jad.2020.07.141 PMC7416772 32818776

[10] Merit Health Wesley. Fear Vs Phobia. https://www.merithealthwesley.com/health-library/14 (accessed on Sep 10, 2023).

[11] GoodRx Health. What’s the Difference Between a Fear vs. a Phobia? https://www.goodrx.com/health-topic/mental-health/fear-vs-phobia (accessed on Sep 10, 2023).

[12] Arora A, Jha AK, Alat P, Das SS (2020). Understanding coronaphobia. Asian J Psychiatry.

[13] Amin S, Mehmood W, Aman-Ullah A, Khan MA. Corona-phobia violated human rights? Impact of COVID-19 on patient’s well-being. Int J Hum Rights Healthc 2022 Dec 8. 10.1108/IJHRH-05-2022-0048.

[14] Kovess-Masfety V, Leray E, Denis L, Husky M, Pitrou I, Bodeau-Livinec F (2016). Mental health of college students and their non-college-attending peers: results from a large French cross-sectional survey. BMC Psychol.

[15] Cvetkovski S, Reavley NJ, Jorm AF (2012). The prevalence and correlates of psychological distress in Australian tertiary students compared to their community peers. Australian New Z J Psychiatry.

[16] Barnett TM, McFarland A, Miller JW, Lowe V, Hatcher SS. Physical and mental health experiences among African American college students. Social Work in 10.1080/19371918.2019.1575308. 10.1080/19371918.2019.1575308 30806178

[17] Khan KS, Mamun MA, Griffiths MD, Ullah I. The mental health impact of the COVID-19 pandemic across different cohorts. Int J Mental Health Addict 2020 Jul 9:1–7. 10.1007/s11469-020-00367-0. 10.1007/s11469-020-00367-0 PMC7347045 32837440

[18] Muyor-Rodríguez J, Caravaca-Sánchez F, Fernández-Prados JS (2021). Covid-19 fear, resilience, social support, anxiety, and suicide among college students in Spain. Int J Environ Res Public Health.

[19] Pomerantz P. Another COVID-19 victim: International education. The Hill. 2020. https://thehill.com/opinion/education/503954-another-covid-19-victim-international-education.

[20] Ma H, Miller C (2021). Trapped in a double bind: Chinese overseas student anxiety during the COVID-19 pandemic. Health Commun.

[21] Elemo AS, Ahmed AH, Kara E, Zerkeshi MK. The fear of COVID-19 and flourishing: assessing the mediating role of sense of control in international students. Int J Mental Health Addict 2021 Apr 5:1–1. 10.1007/s11469-021-00522-1. 10.1007/s11469-021-00522-1 PMC8021294 33841052

[22] Lai AY, Lee L, Wang MP, Feng Y, Lai TT, Ho LM, Lam VS, Ip MS, Lam TH. Mental health impacts of the COVID-19 pandemic on international university students, related stressors, and coping strategies. Front Psychiatry. 2020;1082. 10.3389/fpsyt.2020.584240. 10.3389/fpsyt.2020.584240 PMC7719620 33329126

[23] Pedersen ER, Fitzke RE, Bouskill KE, Sedano A. A qualitative look at the impact of the COVID-19 pandemic on American College Students studying abroad. Frontiers: Interdisciplinary J Study Abroad. 2021;33(3). 10.36366/frontiers.v33i3.602.

[24] Wikipedia. Minorities in Korea. In Wikipedia. https://en.wikipedia.org/wiki/Minorities_in_Korea (accessed on Feb 17, 2024).

[25] Rakushin LA, Hak-Soo Y (2022). Trends and challenges: Chinese students studying at South Korean universities. Asian J Univ Educ.

[26] Malik S, Ullah I, Irfan M, Ahorsu DK, Lin CY, Pakpour AH, Griffiths MD, Rehman IU, Minhas R (2021). Fear of COVID-19 and workplace phobia among Pakistani doctors: a survey study. BMC Public Health.

[27] Monterrosa-Castro A, Redondo-Mendoza V, Mercado-Lara M (2020). Psychosocial factors associated with symptoms of generalized anxiety disorder in general practitioners during the COVID-19 pandemic. J Investig Med.

[28] Heiat M, Heiat F, Halaji M, Ranjbar R, Marvasti ZT, Yaali-Jahromi E, Azizi MM, Hosseini SM, Badri T (2021). Phobia and fear of COVID-19: origins, complications and management, a narrative review. Annali Di Igiene Med Preventiva E Di Comunita.

[29] Ali M, Uddin Z, Banik PC, Hegazy FA, Zaman S, Ambia AS, Siddique M, Bin K, Islam R, Khanam F, Bahalul SM. Knowledge, attitude, practice, and fear of COVID-19: an online-based cross-cultural study. Int J Mental Health Addict 2021 Aug 30:1–6. 10.1007/s11469-021-00638-4. 10.1007/s11469-021-00638-4 PMC8404540 34483782

[30] Hossain MA, Jahid MI, Hossain KM, Walton LM, Uddin Z, Haque MO, Kabir MF, Arafat SY, Sakel M, Faruqui R, Hossain Z (2020). Knowledge, attitudes, and fear of COVID-19 during the Rapid rise period in Bangladesh. PLoS ONE.

[31] Douglas M, Katikireddi SV, Taulbut M, McKee M, McCartney G. Mitigating the wider health effects of COVID-19 pandemic response. BMJ. 2020;369. 10.1136/bmj.m1557. 10.1136/bmj.m1557 PMC7184317 32341002

[32] Khumsaen N, Peawnalaw S. Factors associated with fear of COVID-19 among people living with HIV/AIDS in Suphanburi Province, Thailand. Japan J Nurs Sci 2022 Mar 1:e12480. 10.1111/jjns.12480. 10.1111/jjns.12480 PMC9115060 35229481

[33] Tsao SF, Chen H, Tisseverasinghe T, Yang Y, Li L, Butt ZA (2021). What social media told us in the time of COVID-19: a scoping review. Lancet Digit Health.

[34] Buchanan M. Managing the infodemic (Doctoral dissertation, Nature Publishing Group). 10.1038/s41567-020-01039-5.

[35] O’sullivan R, Burns A, Leavey G, Leroi I, Burholt V, Lubben J, Holt-Lunstad J, Victor C, Lawlor B, Vilar-Compte M, Perissinotto CM (2021). Impact of the Covid-19 pandemic on loneliness and social isolation: a multi-country study. Int J Environ Res Public Health.

[36] Lemenager T, Neissner M, Koopmann A, Reinhard I, Georgiadou E, Müller A, Kiefer F, Hillemacher T (2021). COVID-19 lockdown restrictions and online media consumption in Germany. Int J Environ Res Public Health.

[37] World Health Organization. Call for action: Managing the infodemic. World Health Organization: Geneva, Switzerland. 2020 Dec 11. https://www.who.int/news/item/11-12-2020-call-for-action-managing-the-infodemic.

[38] Moscadelli A, Albora G, Biamonte MA, Giorgetti D, Innocenzio M, Paoli S, Lorini C, Bonanni P, Bonaccorsi G (2020). Fake news and Covid-19 in Italy: results of a quantitative observational study. Int J Environ Res Public Health.

[39] van Der Linden S, Roozenbeek J, Compton J (2020). Inoculating against fake news about COVID-19. Front Psychol.

[40] Apuke OD, Omar B (2021). Fake news and COVID-19: modelling the predictors of fake news sharing among social media users. Telematics Inform.

[41] Zhao B, Kong F, Nam EW. Relationship between eHealth, Perceived Risk, and Phobia of COVID-19 among Chinese University Students in Korea and China. Health & Social Care in the Community. 2023;2023. 10.1155/2023/2755354.

[42] Brørs G, Norman CD, Norekvål TM (2020). Accelerated importance of eHealth literacy in the COVID-19 outbreak and beyond. Eur J Cardiovasc Nurs.

[43] Harnett S (2020). Health literacy, social media and pandemic planning. J Consumer Health Internet.

[44] Oh SH, Lee SY, Han C (2021). The effects of social media use on preventive behaviors during infectious disease outbreaks: the mediating role of self-relevant emotions and public risk perception. Health Commun.

[45] Lee SM, Lee D (2020). Lessons learned from battling COVID-19: the Korean experience. Int J Environ Res Public Health.

[46] Han E, Tan MM, Turk E, Sridhar D, Leung GM, Shibuya K, Asgari N, Oh J, García-Basteiro AL, Hanefeld J, Cook AR (2020). Lessons learnt from easing COVID-19 restrictions: an analysis of countries and regions in Asia Pacific and Europe. Lancet.

[47] CNN. One in five South Koreans have had Covid, as latest wave sees deaths surge. March 24. 2022. https://edition.cnn.com/2022/03/24/asia/south-korea-covid-surge-deaths-intl-hnk/index.html (accessed on April 22, 2022).

[48] REUTERS. S.Korea likely to lift outdoor mask mandate, most COVID curbs this month. https://www.reuters.com/business/healthcare-pharmaceuticals/skorea-likely-lift-outdoor-mask-mandate-most-covid-curbs-this-month-2022-04-01/ (accessed on April 22, 2022).

[49] The Korea Herald. S. Korea begins discussion on ending restrictions as cases fall. http://www.koreaherald.com/view.php?ud=20220411000625 (accessed on April 22, 2022).

[50] FKCCI. [UPDATE - COVID-19]. Sanitary rules in South Korea. https://www.fkcci.com/actualites/n/news/update-covid-19-sanitary-rules-in-south-korea.html (accessed on April 22, 2022).

[51] REUTERS. Shanghai widens COVID testing as other Chinese cities impose curbs. https://www.reuters.com/world/china/shanghai-widens-covid-testing-other-chinese-cities-impose-curbs-2022-04-08/ (accessed on April 28, 2022).

[52] Our World in Data. Coronavirus (COVID-19) Cases. https://ourworldindata.org/covid-cases (accessed on April 28, 2022).

[53] Cahuas A, Marenus MW, Kumaravel V, Murray A, Friedman K, Ottensoser H, Chen W (2023). Perceived social support and COVID-19 impact on quality of life in college students: an observational study. Ann Med.

[54] Aylie NS, Mekonen MA, Mekuria RM. The psychological impacts of COVID-19 pandemic among university students in Bench-Sheko Zone, South-West Ethiopia: a community-based cross-sectional study. Psychology research and behavior management. 2020 Sep 30:813–21. 10.2147/PRBM.S275593. 10.2147/PRBM.S275593 PMC7533263 33061696

[55] Clements JM (2020). Knowledge and behaviors toward COVID-19 among US residents during the early days of the pandemic: cross-sectional online questionnaire. JMIR Public Health Surveillance.

[56] Lee M, Kang BA, You M (2021). Knowledge, attitudes, and practices (KAP) toward COVID-19: a cross-sectional study in South Korea. BMC Public Health.

[57] Zhong BL, Luo W, Li HM, Zhang QQ, Liu XG, Li WT, Li Y (2020). Knowledge, attitudes, and practices towards COVID-19 among Chinese residents during the rapid rise period of the COVID-19 outbreak: a quick online cross-sectional survey. Int J Biol Sci.

[58] Rimal RN, Juon HS (2010). Use of the risk perception attitude framework for promoting breast cancer prevention. J Appl Soc Psychol.

[59] Arpaci I, Alshehabi S, Al-Emran M, Khasawneh M, Mahariq I, Abdeljawad T, Hassanien AE (2020). Analysis of twitter data using evolutionary clustering during the COVID-19 pandemic. Computers Mater Continua.

[60] Arpaci I, Karataş K, Baloğlu M (2020). The development and initial tests for the psychometric properties of the COVID-19 phobia scale (C19P-S). Pers Indiv Differ.

[61] ALJAZEERA. China COVID cases more than double in growing outbreak. https://www.aljazeera.com/news/2022/3/15/china-covid-cases-more-than-double-in-growing-outbreak (accessed on April 12, 2022).

[62] The Wall Street Journal. Lockdowns Spread Across China as Race to Contain Covid-19 Outbreak Intensifies. https://www.wsj.com/articles/lockdowns-spread-across-china-as-race-to-contain-covid-19-outbreak-intensifies-11647266905 (accessed on April 12, 2022).

[63] Korea Disease Control and Prevention Agency. COVID-19. From March 1st, it is converted to manual surveillance, regardless of vaccination, which is the cohabitation of the confirmed person. http://ncov.mohw.go.kr/tcmBoardView.do?contSeq=370385 (accessed on April 12, 2022) (in Korean).

[64] Zhao B, Kong F, Nam EW. Assessing Knowledge, preventive practices, and depression among Chinese university students in Korea and China during the COVID-19 pandemic: An online cross-sectional study. InHealthcare 2021 Apr (Vol. 9, No. 4, p. 433). Multidisciplinary Digital Publishing Institute. 10.3390/healthcare9040433. 10.3390/healthcare9040433 PMC8067962 33917674

[65] Kim HR, Kim EJ (2021). Factors associated with mental health among international students during the COVID-19 pandemic in South Korea. Int J Environ Res Public Health.

[66] Wolf LJ, Haddock G, Manstead AS, Maio GR (2020). The importance of (shared) human values for containing the COVID-19 pandemic. Br J Soc Psychol.

[67] Lu L, Liu J, Yuan YC, Burns KS, Lu E, Li D (2021). Source trust and COVID-19 information sharing: the mediating roles of emotions and beliefs about sharing. Health Educ Behav.

[68] Dutta-Bergman M (2003). Trusted online sources of health information: differences in demographics, health beliefs, and health-information orientation. J Med Internet Res.

[69] National Cancer Institute HINTS Brief 39: Trust in health information sources among American adults. 2019. https://hints.cancer.gov/docs/Briefs/HINTS_Brief_39.pdf.

[70] Chang KC, Strong C, Pakpour AH, Griffiths MD, Lin CY (2020). Factors related to preventive COVID-19 infection behaviors among people with mental illness. J Formos Med Assoc.

[71] Lăzăroiu G, Adams C (2020). Viral panic and contagious fear in scary times: the proliferation of COVID-19 misinformation and fake news. Anal Metaphysics.

[72] Naeem M (2021). Do social media platforms develop consumer panic buying during the fear of Covid-19 pandemic. J Retailing Consumer Serv.

[73] Gao J, Zheng P, Jia Y, Chen H, Mao Y, Chen S, Wang Y, Fu H, Dai J (2020). Mental health problems and social media exposure during COVID-19 outbreak. PLoS ONE.

[74] Jang IC, Choi LJ (2020). Staying connected during COVID-19: the social and communicative role of an ethnic online community of Chinese international students in South Korea. Multilingua.

[75] Ghaderi E, Mahmoodi H, Sharifi Saqqezi P, Ghanei Gheshlagh R, Moradi G, Shokri A, Piroozi B, Mohamadi Bolbanabad A, Ahmadi A (2022). Knowledge, attitudes, practices and fear of COVID-19 among iranians: a quick online survey. Health Soc Care Commun.

[76] Tachfouti N, Slama K, Berraho M, Nejjari C. The impact of knowledge and attitudes on adherence to tuberculosis treatment: a case-control study in a Moroccan region. Pan Afr Med J. 2012; 12(1). http://www.panafrican-med-journal.com/content/article/12/52/full/. PMC3428172 22937192

[77] Ajilore K, Atakiti I, Onyenankeya K (2017). College students’ knowledge, attitudes and adherence to public service announcements on Ebola in Nigeria: suggestions for improving future Ebola prevention education programmes. Health Educ J.

[78] U.S. CHINA RELATIONS. Coronavirus Social Impact: Difficult Choices for Chinese International Students. https://www.ncuscr.org/podcast/coronavirus-chinese-students/ (accessed on May 12, 2022).

[79] Eliot D, the U.S. UVM College of Arts and Sciences College Honors Theses. COVID-19: How a Pandemic Exposed the Vulnerability of International Students in. 2021; 89. https://scholarworks.uvm.edu/castheses/89 (accessed on May 12, 2022).

[80] Seong M, Lee M, Kim I, Kang M (2021). Validation of the Korean Version of the COVID-19 phobia scale (K-C19PS). Int J Environ Res Public Health.

[81] Brick JM. Explorations in non-probability sampling using the web. In Proceedings of the Conference on Beyond Traditional Survey Taking: Adapting to a Changing World, Canada, 29–31 October 2014; Berret Koehler Publishers: Oakland, CA, USA, 2014; pp. 1–6.

