# Supplementary material for: Exploring COVID-19 Phobia among International Chinese College Students in South Korea Before Ending COVID-19 Restrictions
Source: BMC Psychol. 2024 Apr 23;12:222. doi: 10.1186/s40359-024-01718-5 (PMC11036663; doi:10.1186/s40359-024-01718-5)
Supplement: Supplementary file 1 — Supplementary Material 1 [file 40359_2024_1718_MOESM1_ESM.docx]

Coronavirus 19 Phobia Scale (C19P-S) items

**Psychological factors**

1. The fear of coming down with coronavirus makes me very anxious.

2. I am extremely afraid that someone in my family might become infected by the coronavirus.

3. News about coronavirus-related deaths causes me great anxiety.

4. Uncertainties surrounding coronavirus cause me enormous anxiety.

5. The pace that coronavirus has spread causes me great panic.

6. I argue passionately (or want to argue) with people I consider to be behaving irresponsibly in the face of coronavirus.

**Psycho-somatic factors**

1. I experience serious stomachaches out of the fear of coronavirus.

2. I experience serious chest pain out of the fear of coronavirus.

3. I experience tremors due to the fear of coronavirus.

4. I experience sleep problems out of the fear of coronavirus.

5. Coronavirus makes me so tense that I find myself unable to do the thing I previously had no problem doing.

**Economic factors**

1. The possibility of food supply shortage due to the coronavirus pandemic causes me anxiety.

2. The possibility of shortages in cleaning supplies due to the cornavirus pandemic causes me anxiety.

3. I stock food with the fear of coronavirus.

4. After the coronavirus pandemic, I do not feel relaxed unless I constantly check on my supplies at home.

**Social factors**

1. After the coronavirus pandemic, I feel extremely anxious when I see people coughing.

2. After the coronavirus pandemic, I actively avoid people I see sneezing.

3. Following the coronavirus pandemic, I have noticed that I spend extensive periods of time cleaning my hands.

4. The fear of coming down with coronavirus seriously impedes my social relationships.

5. I am unable to curb my anxiety of catching coronavirus from others.
